# Supplementary material for: Distribution and evolution of the LysR-type transcriptional regulators of the Salmonella genus
Source: PLoS One. 2026 Mar 17;21(3):e0344260. doi: 10.1371/journal.pone.0344260 (PMC12994799; doi:10.1371/journal.pone.0344260)
Supplement: Attachment — Submitted filename: Response to Reviewers.docx [file pone.0344260.s004.docx]

September 2025.

Prof. Priyanka Sharma

Editor. PLOS ONE

Dear Prof. Priyanka Sharma

Please find enclosed the manuscript entitled: Distribution and evolution of the LysR-Type transcriptional regulators of the *Salmonella* genus. (PONE-D-25-23217). This work was previously submitted to PLOS ONE and minor revision were suggested. The revised manuscript includes all the suggestions raised by the reviewers and the editor.

In the response letter and in the manuscript, we include all the corrections. The paper is now greatly improved and we are grateful to you and the reviewers for their insightful comments.

I hope that you find the revised paper to be acceptable for publication in PLOS ONE.

Yours sincerely, Ismael Hernández-Lucas, Corresponding Author

Mailing address: Departamento de Microbiología Molecular, Instituto de Biotecnología, Universidad Nacional Autónoma de México, Av. Universidad 2001,

Cuernavaca, Morelos 62210, México. Phone: (52) (777) 329-1627. Fax: (52) (777) 3138673. E-mail: ismael.hernandez@ibt.unam.mx

Ms. No. PONE-D-25-23217

Distribution and evolution of the LysR-Type transcriptional regulators of the *Salmonella* genus. By Dávila S^§^, Gama-Martínez Y^§^, Orozco R_,_ Rivera-Ramírez A Hernández VM and Hernández-Lucas I*. ^§^ These authors contribute equally to this work.

Responses to reviewers in normal font.

1. Is the manuscript technically sound, and do the data support the conclusions?

The manuscript must describe a technically sound piece of scientific research with data that supports the conclusions. Experiments must have been conducted rigorously, with appropriate controls, replication, and sample sizes. The conclusions must be drawn appropriately based on the data presented.

Reviewer #1: Partly

Reviewer #2: Yes

Reviewer #3: Yes

2. Has the statistical analysis been performed appropriately and rigorously?

Reviewer #1: No

Reviewer #2: Yes

Reviewer #3: N/A

3. Have the authors made all data underlying the findings in their manuscript fully available?

The PLOS Data policy requires authors to make all data underlying the findings described in their manuscript fully available without restriction, with rare exception (please refer to the Data Availability Statement in the manuscript PDF file). The data should be provided as part of the manuscript or its supporting information, or deposited to a public repository. For example, in addition to summary statistics, the data points behind means, medians and variance measures should be available. If there are restrictions on publicly sharing data—e.g. participant privacy or use of data from a third party—those must be specified.

Reviewer #1: Yes

Reviewer #2: Yes

Reviewer #3: Yes

4. Is the manuscript presented in an intelligible fashion and written in standard English?

PLOS ONE does not copyedit accepted manuscripts, so the language in submitted articles must be clear, correct, and unambiguous. Any typographical or grammatical errors should be corrected at revision, so please note any specific errors here.

Reviewer #1: Yes

Reviewer #2: Yes

Reviewer #3: No

5. Review Comments to the Author

# Comments of reviewer 1

Please use the space provided to explain your answers to the questions above. You may also include additional comments for the author, including concerns about dual publication, research ethics, or publication ethics. (Please upload your review as an attachment if it exceeds 20,000 characters)

Reviewer #1: 1) The Materials and Methods section briefly states that LTTRs were obtained from the Salmonella CT18 genome. However, the manuscript does not clarify the methodology used for their identification. The authors should elaborate on the approach: What specific tools, databases, or bioinformatic pipelines were used? Overall, the Methods section can be more descriptive.

Response: The new version of the manuscript includes subtitles, tools, databases, genome accessions as well as a new understandable explanation of the material and methods used in this study. Thus, we modify the complete section of materials and methods as you suggested.

Materials and methods.

LTTR identification in *Salmonella* and bacterial genomes.

The PROSITE database was used to obtain LTTRs from the *S*. Typhi CT18 genome (ID 220341) (https://prosite.expasy.org/PS50931). The conserved domain accessions PF0126 and PF03466 were applied to verify the *S*. Typhi LTTR authenticity. Furthermore ClustalW (https://www.genome.jp/tools-bin/clustalw) sequence alignment showed that all the LTTRs identified in *S*. Typhi CT18 genome contain the winged helix DNA binding domain, the linker helix domain and periplasmic binding domain characteristic of the LTTR family (9). The resultant 41 LTTRs of *S*. Typhi (Fig. 1) were used as input query sequences to find orthologous proteins in prokaryotic genomes contained in the UniProt database. LTTR sequences were selected only if they shared ≥90% amino acid identity with the LTTRs of *S*. Typhi. Using this filtering strategy *S*. Typhi LTTR orthologs were identified in 33 genomes of *Salmonella enterica* subspecies *enterica*, in one genome of *Salmonella bongori* and in one genome each of the subspecies *arizonae, diarizonae, houtenae, indica* and *salamae*. Thus, *S*. Typhi LTTRs orthologous sequences in 39 genomes belonging to the *Salmonella* genus were identified. Orthologous LTTRs sequences of *S*. Typhi CT18 from other bacterial phyla including *Enterobacteriaceae* and *Phyllobacteriaceae*. were also obtained using this filtering strategy. The LTTR data obtained was used to generate Figs.1- 4.

LTTR distribution in *Salmonella* and bacterial genomes.

A phylogenetic tree of species based on NCBI taxonomy of ribosomal proteins using the software PhyloT (https://phylot.biobyte.de/) was constructed to facilitate the visualization of LTTR distribution in *Enterobacteriaceae* and *Phyllobacteriaceae*. The resulting tree was manipulated, annotated and visually enhanced using of phylogenetic tool iTOL (https://itol.embl.de/). In the supplemental material we include the Taxon Id of the genomes utilized to generate Figure 3.

Phylogenetic relationships of bacteria based on the LTTR CysB and ribosomal proteins.

To determine the phylogeny of the LTTR CysB in the broader context of bacterial species, a phylogenetic tree of these organisms based on CysB proteins was generated. A second phylogenetic tree of bacterial species based on ribosomal protein sequences using data from the Genome Taxonomy Database (GTDB) was also constructed. Specifically, a phylogenetic reconciliation was carried out between the CysB tree and the ribosomal species-level tree. For all these analyses, ClustalW (https://www.genome.jp/tools-bin/clustalw) was employed to perform multiple sequence alignments, reconstruct, and compare the CysB and ribosomal protein phylogenies. The final visualization of the phylogenetic reconciliation analysis between CysB and ribosomal proteins (Fig.4) was created through a combination of phylogenetic tools, including iTOL for tree editing, Inkscape (https://inkscape.org/) for image modification and refinement, and R (https://www.r-project.org/) for additional data handling and figure generation.

2) Figure 1 needs improved visualization. The authors may consider redesigning this figure to enhance readability, perhaps by using a color-coded heatmap or a presence/absence matrix with clear gridlines. This would help the audience better interpret and appreciate the comparative distribution of LTTRs across strains or species.

Response: Figure 1 has been redesigning. The new figure includes colors and clear gridlines as you suggested

3) I think a graphical presentation of the LTTR distribution will help the manuscript. The text refers multiple times to the common versus exclusive presence of LTTRs among strains. This information would be more impactful if presented graphically. The authors could consider including pie charts, bar plots, or Venn diagrams to summarize the shared and unique LTTRs across the dataset.

Response: As you suggested, a graphical representation (Fig 2) of the LTTR distribution in the *Salmonella* genus was included in the new version of the manuscript

4) The claim regarding CysB as an evolutionary marker appears somewhat overstated. Unlike ribosomal proteins, which are universally conserved and widely used in phylogenetic studies, CysB is not present across all bacterial taxa and seems to be largely restricted to Enterobacteriaceae. At best, CysB may serve as a functional evolutionary marker within specific niches or lineages.

Response: In the new version of the manuscript, we modify the text about CysB as evolutionary marker, instead we added this paragraph as you suggested

We propose to the LTTR CysB as an additional tool to be used in molecular phylogenetic studies in *Enterobacteriaceae.*

5) Moreover, regulatory genes such as CysB are often subject to horizontal gene transfer, which can confound phylogenetic interpretations. What are authors view on this. Maybe the authors should address these caveats and clarify whether their use of "evolutionary marker" refers to functional divergence rather than strict lineage tracing.

Response: We are agreeing with your comment, thus we just stated the role of CysB as an additional tool to be used in molecular phylogenetic studies in *Enterobacteriaceae.*

# Comments of reviewer 2

Reviewer #2: This is a very interesting study in LysR-Type transcriptional regulators (LTTRs). Further research is needed, for better understanding the role of these proteins, in order to prevent and control infections caused by Salmonella, in adults and children.

Response: You are right, in this regard our group currently are working in the LTTR characterization for better understanding the role of these proteins, in order to prevent and control infections caused by *Salmonella* in adults and children.

# Comments of reviewer 3

Reviewer #3: Dear Authors,

Thank you for submitting your manuscript to PLoS ONE. Your study presents an insightful and methodologically strong investigation into the evolution and distribution of LTTRs in Salmonellae and other bacterial genera. The identification of core and accessory LTTR pools has meaningful implications for understanding bacterial adaptability and gene regulation. However, to improve the manuscript, kindly consider the following;

• Please revise for grammatical accuracy and clarity, particularly in the abstract, introduction, and discussion. Some phrases are meaningless because of either too short or too long to understand.

Response. A native speaker English has reviewed the new version of the manuscript.

• Avoid repetition between results and discussion; use the latter to provide synthesis and implications.

Response. Corrected as you suggested

• Add clarifications to your methods, such as genome accession details and criteria for LTTR classification.

Response. The new version of the manuscript includes subtitles, tools, databases, genome accessions and criteria for LTTR classification as well as and understandable explanation of the material and methods used in this study. Thus, we modify the complete section of materials and methods as you suggested.

• The name of bacterial spp. should be written in standard way of being italic, however, the same is missing in the text, this needs to be carefully revised.

Response. In the new version of the manuscript all the bacterial spp are correctly written as you suggested.

Once these revisions are made, the manuscript will be suitable for publication. Thank you again for your contribution.

Response: All your suggestions and corrections of other two reviewers and of the editor were addressed. Thus, the new version of the manuscript is now greatly improved, and we are grateful to you and the reviewers for their insightful comments.
