# Supplementary material for: Distribution and evolution of the LysR-type transcriptional regulators of the Salmonella genus
Source: PLoS One. 2026 Mar 17;21(3):e0344260. doi: 10.1371/journal.pone.0344260 (PMC12994799; doi:10.1371/journal.pone.0344260)
Supplement: Attachment — Submitted filename: Detailed_comments.docx [file pone.0344260.s005.docx]

The authors present a study on the establishment of a *Salmonella* LTTR core, which is an interesting and potentially valuable contribution. The revised version shows substantial improvement compared with the previous submission, and several analyses appear promising. Before the manuscript can be considered for publication, however, a number of clarifications, linguistic improvements, and additional references are required to strengthen the scientific arguments and ensure clarity. The manuscript would also benefit from careful language editing, ideally with input from a native English speaker, as several sentences contain grammatical errors or unclear phrasing.

Below are specific comments that should be addressed in the revised draft:

**Major and Minor Comments**

1. **Introduction**
   Please provide a clear explanation of *cysB* and its biological role in the introduction. The introduction should also more thoroughly discuss the role and significance of LTTRs in *Salmonella*. The rationale for selecting *cysB* is not clearly articulated and should be stated explicitly.
2. **Lines 29–30**
   This sentence appears incomplete. Please revise to clearly convey the intended meaning.
3. **Lines 70–71**
   Please rewrite this sentence for clarity and correct grammar.
4. **Lines 94–97**
   The filtering strategy used in the analysis needs to be described more clearly and in greater detail.
5. **Lines 100–102**
   Please revise this statement. A phylogenetic tree is not typically used to assess the distribution of LTTRs.
6. **Lines 104–105**
   Please check and correct the grammar.
7. **Line 114**
   The phrase “*CysB and ribosomal protein phylogenies*” is unclear. The term *phylogeny* may not be the most appropriate here. Please revise for scientific accuracy.
8. **Lines 115–118**
   The methods used for phylogenetic tree generation, including bootstrap values and all relevant parameters, should be described more thoroughly.
9. There are several existing studies reporting ribosomal phylogenetic trees. Please cite the relevant literature and discuss how your findings compare, particularly regarding clade structure and similarity.
10. Please discuss why LTTRs appear to be present in only specific serotypes and relate these observations to existing literature on LTTR expression, bacterial biology, and pathogenicity.
11. Since LTTR expression was identified in public databases, consider evaluating whether LTTR profiles correlate with antimicrobial susceptibility, if such metadata are available.
12. **Lines 124–126**
    You mention that the primary reason for studying LTTRs is the high morbidity of *Salmonella Typhi*. Please clarify this rationale and specifically state why LTTRs were chosen for this investigation.
13. **Lines 147–148**
    Please provide references supporting the statement that certain subspecies adapt to hosts or environments with a reduced number of LTTRs.
14. **Lines 149–152**
    This statement seems overly general. Please provide scientific justification explaining why LTTR reduction might occur.
15. **Lines 146–157**
    Please provide references and additional detail regarding the hypothetical proteins mentioned. Clarify whether the LTTR core differs among species or serotypes.
16. **Lines 163–164**
    Are LTTR cores or cassettes present in other bacterial taxa? If so, please describe which genes are involved and how they compare to the *Salmonella* LTTR core, with appropriate references.
    Additionally, discuss whether the specific LTTR distribution might contribute to serotype-specific pathogenic behaviors.
17. **Lines 185–186**
    Please clarify the meaning of “*Salmonella taxonomic group*.”
18. **Lines 190–191**
    The heading “*LTTRs of the Salmonella genus are present in Enterobacteriaceae Phyllobacteriaceae*” is confusing, since *Salmonella* itself belongs to Enterobacteriaceae. Consider revising to something like “Presence of LTTRs in Other Enterobacteriaceae Members” to improve clarity.
    **Lines 192–194:** Please rewrite this section to convey the findings clearly.
19. **Lines 202–203**
    Please clarify what is meant by “between 13 and 14.”
20. **Line 206**
    Please revise the phrase “common evolutionary story of LTTR evolution” for clarity and scientific accuracy.
21. **Lines 211–213**
    Please rewrite this statement for clarity and list the pathogens in a scientifically appropriate manner.
22. **Lines 214–216**
    Please specify which bacteria share this particular order of LTTRs, and clarify whether this applies to all bacteria listed in lines 213–214.
23. **Lines 218–221**
    The sentence contains repeated phrasing (“were obtained”). Please revise for clarity and grammatical correctness.
24. **Lines 221–222**
    Please refer to the correct table number in the text.
25. **Line 228**
    The term “Essential genome core” is not standard. Please use established terminology from phylogenomics.
26. **Line 236**
    Please clarify how the presence of both variable and conserved regions is “fundamental for biological processes,” as stated.
27. **Line 239**
    Please correct the grammar.
28. **Lines 243–245**
    Are there references showing that any specific gene can serve as a phylogenetic classifier? In *Salmonella*, even MLST using seven housekeeping genes has limited discriminatory power. Please justify the claim that *cysB* can be used in this way, including supporting references or comparative examples.
29. **Line 247**
    Please clarify the meaning of “Reconciliation” in this context.
30. **Line 251**
    Please provide the species and accession numbers for sequences downloaded from each genus.
31. Please clarify the overall conclusion and practical relevance of your findings related to *cysB*.
32. The manuscript touches on transcriptional regulation by LTTRs but does not clearly explain its biological implications. Please expand on what LTTRs control and why this is relevant.
33. LTTRs are important for evolution and transcriptional regulation; however, this is not reflected in the discussion. Please elaborate and cite relevant literature.
34. **Lines 275–278**
    The statement is unclear regarding whether LTTR number is dependent or independent of genome size. Please clarify and cite supporting references.
35. **Line 298**
    When discussing genetic redundancy, please provide quantitative or descriptive information on how similar the LTTRs are.
36. **Line 302**
    If LTTRs similar to *Salmonella* LTTRs are present in other species, please clarify this section to avoid implying that LTTRs are exclusive to *Salmonella*. Revise the wording to accurately describe how LTTRs are shared across species and how they differ.
37. **Supplementary Table 1**
    Please clarify whether the listed numbers represent genome accession IDs.
38. **Figures** are currently low resolution and difficult to evaluate. Please provide high-quality, high-resolution figures in the revised submission.
