# Supplementary material for: Distribution and evolution of the LysR-type transcriptional regulators of the Salmonella genus
Source: PLoS One. 2026 Mar 17;21(3):e0344260. doi: 10.1371/journal.pone.0344260 (PMC12994799; doi:10.1371/journal.pone.0344260)
Supplement: Attachment — Submitted filename: Response_to_reviewers2.docx [file pone.0344260.s006.docx]

+The authors present a study on the establishment of a *Salmonella* LTTR core, which is an interesting and potentially valuable contribution. The revised version shows substantial improvement compared with the previous submission, and several analyses appear promising. Before the manuscript can be considered for publication, however, a number of clarifications, linguistic improvements, and additional references are required to strengthen the scientific arguments and ensure clarity. The manuscript would also benefit from careful language editing, ideally with input from a native English speaker, as several sentences contain grammatical errors or unclear phrasing.

Below are specific comments that should be addressed in the revised draft:

**Major and Minor Comments**

1. **Introduction**
   Please provide a clear explanation of *cysB* and its biological role in the introduction. The introduction should also more thoroughly discuss the role and significance of LTTRs in *Salmonella*. The rationale for selecting *cysB* is not clearly articulated and should be stated explicitly. **Corrected as you suggested.**
2. **Lines 29–30**
   This sentence appears incomplete. Please revise to clearly convey the intended meaning. **Corrected as you suggested**
3. **Lines 70–71**
   Please rewrite this sentence for clarity and correct grammar. **Corrected as you suggested**
4. **Lines 94–97**
   The filtering strategy used in the analysis needs to be described more clearly and in greater detail. **Corrected as you suggested**
5. **Lines 100–102**
   Please revise this statement. A phylogenetic tree is not typically used to assess the distribution of LTTRs. **This phrase was eliminated as you suggested**
6. **Lines 104–105**
   Please check and correct the grammar. **Corrected as you suggested**
7. **Line 114**
   The phrase “*CysB and ribosomal protein phylogenies*” is unclear. The term *phylogeny* may not be the most appropriate here. Please revise for scientific accuracy. **This phrase was eliminated as you suggested**
8. **Lines 115–118**
   The methods used for phylogenetic tree generation, including bootstrap values and all relevant parameters, should be described more thoroughly. **Corrected as you suggested**
9. There are several existing studies reporting ribosomal phylogenetic trees. Please cite the relevant literature and discuss how your findings compare, particularly regarding clade structure and similarity. **The comparison of ribosomal and CysB phylogenetic trees was eliminated, since ribosomal proteins are widely distributed phylogenetic marker in most of the bacteria reported so far, in the case of CysB it is only present in few bacteria taxa and in this work, we show that CysB is only able to** **separate clades at the genus level in the *Enterobacterial* order. Thus, CysB can be considered as an additional phylogenetical tool only in *Enterobacteriales****.*

Please discuss why LTTRs appear to be present in only specific serotypes and relate these observations to existing literature on LTTR expression, bacterial biology, and pathogenicity. **The LTTR numbers are variable in distinct pathogens, maybe the duplication events and the maintenance of these duplicates in specific *Salmonella* serotypes are the reason because the LTTR number is different in this genus. Another explanation is that the number of LTTRs in *Salmonella* varies because their regulatory cascades involve multiple LTTRs, in this regard we previously publish that multiple LTTRs are involved in specific biological process such as biofilm formation, bile resistance and porin synthesis (**[**https://doi.org/10.1371/journal.pone.0338130**](https://doi.org/10.1371/journal.pone.0338130)**), even more, in other organisms also multiple LTTRs are involved in swarming, tolerance to copper, zinc and to oxidative stress**  [**https://doi.org/10.1016/j.ijbiomac.2024.132315**](https://doi.org/10.1016/j.ijbiomac.2024.132315)**. This discussion is included in the new version of the manuscript as you suggested**

11. **Lines 124–126**
    You mention that the primary reason for studying LTTRs is the high morbidity of *Salmonella Typhi*. Please clarify this rationale and specifically state why LTTRs were chosen for this investigation. **Corrected as you suggested**
12. **Lines 147–148**
    Please provide references supporting the statement that certain subspecies adapt to hosts or environments with a reduced number of LTTRs. This statement seems overly general. Please provide scientific justification explaining why LTTR reduction might occur. **We are agree with you, the statement is very general, that's why it was eliminated, and the new version of the manuscript only describe the results obtained.**
13. **Lines 146–157**
    Please provide references and additional detail regarding the hypothetical proteins mentioned**. References about the hypothetical proteins mentioned were included in the new version of the manuscript as you suggested.** Clarify whether the LTTR core differs among species or serotypes. **The LTTR core is the same in all the serotypes described, this information was clarified in the new version of the manuscript as you suggested**
14. **Lines 163–164**
    Are LTTR cores or cassettes present in other bacterial taxa? If so, please describe which genes are involved and how they compare to the *Salmonella* LTTR core, with appropriate references. **Your question is the subject of another research project. In this manuscript, we only focus on the *Salmonella* genus.**
15. Additionally, discuss whether the specific LTTR distribution might contribute to serotype-specific pathogenic behaviors. **The LTTR distribution does not correspond to pathogenic behavior, since in *S*. Typhi LTTR number is 41 and in other human pathogen such as *Mycobacterium tuberculosis* the LTTR number decreased to only four and in *Helicobacter pylori* LTTRs are absent. These data show that even in intracellular human pathogens the LTTR number is different. Determining why there are different numbers of LTTRs in the genus *Salmonella,* is a prosperous avenue of research that will undoubtedly generate novel and relevant information regarding the biology of these transcriptional factors in *Salmonella.* This information as well as other data were included in the new version of the manuscript, as you suggested.**
16. **Lines 185–186**
    Please clarify the meaning of “*Salmonella taxonomic group*.” **This phrase was removed to avoid confusion.**
17. **Lines 190–191**
    The heading “*LTTRs of the Salmonella genus are present in Enterobacteriaceae Phyllobacteriaceae*” is confusing, since *Salmonella* itself belongs to Enterobacteriaceae. Consider revising to something like “Presence of LTTRs in Other Enterobacteriaceae Members” to improve clarity. **The heading was modified as you suggested.**
18. **Lines 192–194:** Please rewrite this section to convey the findings clearly. **Corrected as you suggested**
19. **Lines 202–203**
    Please clarify what is meant by “between 13 and 14.” **We eliminate between 13 and 14 to avoid confusion,** **as you suggested**
20. **Line 206**
    Please revise the phrase “common evolutionary story of LTTR evolution” for clarity and scientific accuracy. **We remove this phrase to avoid confusion, as you suggested**
21. **Lines 211–213**
    Please rewrite this statement for clarity and list the pathogens in a scientifically appropriate manner. **We remove part of this phrase to avoid confusion**
22. **Lines 214–216**
    Please specify which bacteria share this particular order of LTTRs, and clarify whether this applies to all bacteria listed in lines 213–214. **We modify this paragraph for clarity, as you suggested.**
23. **Lines 218–221**
    The sentence contains repeated phrasing (“were obtained”). Please revise for clarity and grammatical correctness. **Were obtained was eliminated, and the new version of the manuscript only contain once time were obtained**
24. **Lines 221–222**
    Please refer to the correct table number in the text. **Corrected as you suggested**
25. **Line 228**
    The term “Essential genome core” is not standard. Please use established terminology from phylogenomics. **The subtitle was rewrite in the new version of the manuscript**
26. **Line 236**
    Please clarify how the presence of both variable and conserved regions is “fundamental for biological processes,” as stated. **This part of the paragraph was eliminated to avoid confusion.**
27. **Line 239**
    Please correct the grammar**.** **Corrected as you suggested.**
28. **Lines 243–245**
    Are there references showing that any specific gene can serve as a phylogenetic classifier? In *Salmonella*, even MLST using seven housekeeping genes has limited discriminatory power. Please justify the claim that *cysB* can be used in this way, including supporting references or comparative examples. **You are right. Therefore, we only mention that CysB orthologous sequences separate clades at the genus level, suggesting that CysB has evolved in parallel with the corresponding lineage. Thus, CysB can be considered as an additional phylogenetical tool in the *Enterobacteriales* order**
29. **Line 247**
    Please clarify the meaning of “Reconciliation” in this context. **We remove the comparison of ribosomal proteins and CysB. Therefore, the word reconciliation was eliminated**
30. **Line 251**
    Please provide the species and accession numbers for sequences downloaded from each genus. **The accession numbers of the sequences utilized in this work were provided in the supplementary table 1**
31. Please clarify the overall conclusion and practical relevance of your findings related to *cysB*. **Corrected as you suggested.**
32. The manuscript touches on transcriptional regulation by LTTRs but does not clearly explain its biological implications. Please expand on what LTTRs control and why this is relevant. **Corrected as you suggested.**
33. LTTRs are important for evolution and transcriptional regulation; however, this is not reflected in the discussion. Please elaborate and cite relevant literature. **Corrected as you suggested.**
34. **Lines 275–278**
    The statement is unclear regarding whether LTTR number is dependent or independent of genome size. Please clarify and cite supporting references. **Corrected as you suggested**
35. **Line 298**
    When discussing genetic redundancy, please provide quantitative or descriptive information on how similar the LTTRs are. **We eliminated the phrase genetic redundancy to avoid confusion**
36. **Line 302**
    If LTTRs similar to *Salmonella* LTTRs are present in other species, please clarify this section to avoid implying that LTTRs are exclusive to *Salmonella*. Revise the wording to accurately describe how LTTRs are shared across species and how they differ. **Corrected as you suggested**
37. **Supplementary Table 1**
    Please clarify whether the listed numbers represent genome accession IDs. **Corrected as you suggested**
38. **Figures** are currently low resolution and difficult to evaluate. Please provide high-quality, high-resolution figures in the revised submission. **Corrected as you suggested**
